# Validation of a commercially available test that enables the quantification of the numbers of CGG trinucleotide repeat expansion in *FMR1* gene

**DOI:** 10.1371/journal.pone.0173279

**Published:** 2017-03-09

**Authors:** Grace X. Y. Lim, Minli Yeo, Yvonne Y. Koh, Tri Indah Winarni, Indhu-Shree Rajan-Babu, Samuel S. Chong, Sultana M. H. Faradz, Ming Guan

**Affiliations:** 1 The BioFactory Pte Ltd, Singapore, Singapore; 2 Division of Human Genetics, Center for Biomedical Research, Faculty of Medicine, Diponegoro University, Semarang, Indonesia; 3 Department of Paediatrics, Yong Loo Lin School of Medicine, National University of Singapore, Singapore, Singapore; 4 Khoo Teck Puat–National University Children’s Medical Institute, National University Health System, Singapore, Singapore; 5 Department of Laboratory Medicine, National University Hospital, Singapore, Singapore; Cedars-Sinai Medical Center, Maxine-Dunitz Neurosurgical Institute, UNITED STATES

## Abstract

In the present study, we evaluated a commercially available TP-PCR-based assay, the FastFraX^TM^ *FMR1* Sizing kit, as a test in quantifying the number of CGG repeats in the *FMR1* gene. Based on testing with well characterized DNA samples from Coriell, the kit yielded size results within 3 repeats of those obtained by common consensus (n = 14), with the exception of one allele. Furthermore, based on data obtained using all Coriell samples with or without common consensus (n = 29), the Sizing kit was 97.5% in agreement with existing approaches. Additionally, the kit generated consistent size information in repeatability and reproducibility studies (CV 0.39% to 3.42%). Clinical performance was established with 198 archived clinical samples, yielding results of 100% sensitivity (95% CI, 91.03% to 100%) and 100% specificity (95% CI, 97.64% to 100%) in categorizing patient samples into the respective normal, intermediate, premutation and full mutation genotypes.

## Introduction

Fragile X syndrome (FXS) and its associated disorders are inheritable genetic diseases attributed to a trinucleotide CGG repeat expansion in the 5’ untranslated region of the *fragile X mental retardation 1* (*FMR1*) gene. There are four allelic classes based on the size of the CGG repeat in the *FMR1* gene, including: Normal (NL), with 5 to 44 repeats; Intermediate (IM), with 45 to 54 repeats; Premutation (PM), with 55 to 200 repeats; and Full mutation (FM), with over 200 repeats [1,2]. FM alleles are also characterized by aberrant hypermethylation of the *FMR1* promoter and silencing of the *FMR1* gene [1,2].

Consequently, in a diagnostic setting it is important to not only detect presence of the CGG expansion, but to also determine its size and methylation status. While many accepted fragile X testing methods are available, there is no single approach that can characterize all aspects of *FMR1* expansions, especially when mosaicism is taken into consideration [1]. Over the past decades, different approaches with their own utilities have been developed. Current ACMG guidelines recommend a combined approach whereby Southern blot is always performed alongside traditional PCR, the only exception being when newer repeat-primed PCR methods are used [1]. While Southern blot is regarded as the current gold standard, it is notoriously labour-intensive, time-consuming, and requires large amounts of DNA. Furthermore, the workflow is not optimized for high-throughput testing, as the number of samples that can be processed simultaneously is limited [3,4]. An alternative, simpler approach is traditional PCR, which utilizes primers targeting sequences flanking the CGG repeat region [4–6]. However, this method is ineffective in amplifying large CGG repeat expansions with high GC content, resulting in non-amplification of large PM or FM alleles [4–6]. As a result, novel PCR designs were explored. Eventual success was found in the form of triplet repeat primed PCR (TP-PCR), which targets the *FMR1* CGG triplet repeats directly [3, 7–10].

In this regard, the single-tube PCR developed by Chen *et al*. (2010) [3] appears to be ideal. This method is reportedly able to generate information on the number of CGG repeats, allele zygosity, and AGG spacers. It incorporates primers targeting both the flanking (gene-specific) and the triplet repeat regions. The two sets of PCR products are then analysed via capillary electrophoresis (CE). The full length gene-specific amplicons derived from the flanking primers are a critical and integrated part of the method for quantifying the CGG repeats, especially when FM specimens are involved. However, this approach also claimed to generate adequate size information without the need to amplify the full length of the *FMR1* CGG repeat region [3]. Incidentally, various approaches coupling TP-PCR with CE, similar in principle to the method described here were developed by Lyon *et al*. (2010) [8] and Hantash *et al*. (2010) [11]_._ These two approaches are similar in that they omit the opposing gene-specific primers targeting the flanking regions. Yet, they differ in the information reported. The former yields the size information only to approximately 55 CGG repeats, and can be used only for screening purposes; while the latter provides only a qualitative assessment of the allelic status, and cannot be used for repeat sizing.

In the present study, we validate the Biofactory’s FastFraX^TM^ *FMR1* Sizing Kit (FastFraX^TM^ SZ kit), which utilises an assay based on the principle described in Warner *et al*. (1996) [7] and with a design similar to Teo *et al*.’s (2012) [12] and Rajan-Babu *et al*.’s (2015) [10] approaches. The assay comprises a direct triplet repeat primed polymerase chain reaction (dTP-PCR) modified from Rajan-Babu *et al*. (2015) [10], coupled with CE for quantifying the number of CGG repeats (i.e. sizing). Our study aims to verify the accuracy and effectiveness of the FastFraX^TM^ SZ kit as a tool for sizing CGG repeats in the *FMR1* gene, and as a follow-up test for the reference and clinical samples found to have expansions by the first line PCR-only screening test as described in Lim *et al*. (2014) [9].

## Materials and methods

### DNA samples

Twenty-nine individual cell line-derived DNA reference samples with CGG repeats of different lengths were acquired from Coriell Institute (Coriell Cell Repositories, Camden, NJ; Table 1). The study included testing the FastFraX^TM^ SZ kit’s analytic sensitivity, analytic specificity, and consistency. The genotypes of all Coriell DNA samples tested in this manuscript are listed in Table 1. All 29 samples (Table 1) were used in an initial blinded small scale study to evaluate performance of the FastFraX^TM^ SZ kit.

**Table 1:** CGG repeat sizes of genomic DNA samples from Coriell Cell Repositories and as determined in the accuracy study of the FastFraX^TM^ SZ kit.

Coriell Sample ID	Genotype	No. of CGG Repeats					
		Expected (X)			FastFraX^TM^ SZ kit (Y)	Difference (Y-X)	
			Coriell	Wilson *et al*. [13] and other studies		Coriell	Wilson *et al*. [13] and other studies
***Samples tested by Wilson et al*. [13], *with consensus (n = 14)***							
*Male*							
NA20244	NL		41	41	41	0	0
NA20232	IM		46	46	46	0	0
NA20230	IM		53	53, 54^a^	54	+1	+1, +0^a^
CD00014	PM		56	56	56	0	0
NA20231	PM		76	76, 78^a^	78	+2	+2, +0^a^
NA06892	PM		93	86, 93^a^	93	0	+7, 0^a^
NA20233	PM		117	117, 119^a^	120	+3	+3, +1^a^
*Female*							
NA07538	NL	Allele 1	29	29	29	0	0
		Allele 2	29	29	29	0	0
NA20243	NL	Allele 1	29	29	29	0	0
		Allele 2	41	41	41	0	0
NA20235	IM	Allele 1	29	29	29	0	0
		Allele 2	45	45	45	0	0
NA20234	IM	Allele 1	31	31	26	-5	-5
		Allele 2	46	46	46	0	0
NA20236	IM	Allele 1	31	31	31	0	0
		Allele 2	53	53, 54^a^	54	+1	+1, +0^a^
NA20242	PM	Allele 1	30	30	30	0	0
		Allele 2	73	73, 74^a^, 73/105^b^	105	+32	+32, +31^a^, 0^b^
NA20240	PM	Allele 1	30	30	30	0	0
		Allele 2	80	80, 82^a^, 81^b^	83	+3	+3, +1^a^, +2^b^
***Samples tested by Wilson et al*. [13], *without consensus (n = 5)***							
*Male*							
NA06906	PM		96	NC, 101^a^	101	+5	NC, 0^a^
NA20237	PM		100–104	NC, 100/137^a^	139	+35	NC, +2^a^
NA06891	PM		118	NC, 120^a^, 119^b^	121	+3	NC, +1^a^, +2^b^
*Female*							
NA20241	PM	Allele 1	30	29, 30^a^	29	0	0, -1^a^
		Allele 2	93–110	NC, 91^a^	125	+15	NC, +34^a^
NA20239	PM	Allele 1	20	20, 21^a^	20	-3	0, -1^a^
		Allele 2	183–193	NC, 200^a^, 198/>200^b^	>200	+7	NC, N/A^a^^,^^b^
***Other samples not tested by Wilson et al*. [13] *(n = 10)***							
*Male*							
NA06890	NL		30	30^b^	30	0	0^b^
NA06852	FM		>200	>200^b^	>200	N/A	N/A^b^
NA06897	FM		477	>200^b^	>200	N/A	N/A^b^
NA07862	FM		501–550	>200^a^^,^^b^	>200	N/A	N/A^a^^,^^b^
NA04025	FM		645	>200^a^^,^^b^	>200	N/A	N/A^a^^,^^b^
NA09237	FM		931–940	>200^a^	>200	N/A	N/A^a^
*Female*							
NA13664	IM	Allele 1	28	30^a^^,^^b^	28	0	-2^a^^,^^b^
		Allele 2	49	51^b^, 52^a^	52	+3	+1^b^, 0^a^
NA06896	PM	Allele 1	23	23^b^	23	0	0^b^
		Allele 2	95–140	113/133-138/155/175/198/ >200^b^	183	+43	-17^b^
NA07537	FM	Allele 1	29	29^a^^,^^b^	29	0	0^a^^,^^b^
		Allele 2	>200	>200^a^^,^^b^	>200	N/A	N/A^a^^,^^b^
NA05847	FM	Allele 1	21	20^a^^,^^b^	20	-1	0^a^^,^^b^
		Allele 2	650	>200^a^^,^^b^	>200	N/A	N/A^a^^,^^b^

^a^ Data from Juusola *et al*. (2012) [14].

^b^ Data from Chen *et al*. (2010) [3].

NC: No consensus.

N/A: Not applicable, as the FastFraX^TM^ SZ kit reports all FM as >200 repeats. Hence, difference in repeat size is not calculated.

The analytic specificity of the FastFraX^TM^ SZ kit was evaluated by using a DNA sample (NA23378 from Coriell Cell Repositories, Camden, NJ) with no direct relevance to Fragile X to test for potential interference. NA23378 harbors a CTG repeat expansion in the dystrophia myotonica-protein kinase (*DMPK*) gene.

To evaluate the sensitivity of the FastFraX^TM^ SZ kit in detecting mosaicism, a male NL sample (NA06890; 30 CGG) was mixed with a male PM sample (NA20233; 120 CGG) or an FM sample (NA04025; >200 CGG) to create simulated mosaic content of different concentrations. These samples were mixed in various proportions, yet maintained at a total DNA input of 100 ng per reaction. The resulting simulated mosaic samples contained 1%, 2.5%, 5%, 10%, 20%, 50% and 100% of the larger expanded sample (PM or FM). Another simulated mosaic sample containing both PM and FM alleles was also created in a similar fashion, with the amount of the FM allele varied at 1%, 2.5%, 5%, 10%, 20%, 50% and 100% of the total DNA input. Electropherograms were analyzed to determine the reportable repeat size of the larger allele that is present in the sample.

### DNA samples from clinical archive

The clinical performance of the FastFraX^TM^ SZ kit was further validated using 198 archived clinical samples, originally obtained with informed consent from a population with intellectual disabilities from several special schools and institutions in Java Island, Indonesia. These genomic DNA samples had been isolated from whole blood using salting out methods as described [15], with slight modifications. The samples had also been characterised for *FMR1* CGG repeat length using a combination of the following methods: a two-primer conventional (flanking) PCR [16] followed by fragment length analysis on an ABI Prism 3730 DNA Analyzer (Life Technologies) with the Genemapper software (Ver 4.0, Apache), or Southern blot analysis [17]. For samples with NL, IM or PM alleles, exact CGG repeat size was determined using a combination of PCR and fragment analysis. For samples with results indicative of a FM, or for female samples that produced a single PCR product, Southern blot analysis was performed to confirm the FM or to differentiate between female heterozygous and homozygous samples [17]. In a few cases, this confirmation was based on TP-PCR as previously described [18]. Genomic DNA concentrations were re-quantified with a NanoVue Plus spectrophotometer (GE Healthcare). Usage of these archived clinical samples for the present study was approved by the Health Research Ethical Commsittee of the Faculty of Medicine, Diponegoro University, and Dr. Kariadi Hospital, Indonesia. All samples were stored at -20°C until use.

### FastFraX^TM^ FMR1 SZ kit direct triplet repeat primed PCR and capillary electrophoresis analysis

The FastFraX^TM^ SZ kits (labelled “For Research Use” (RUO)) were obtained from the BioFactory Pte Ltd, Singapore. The kit was developed based on a previous study by Rajan-Babu *et al*. [10]. It utilizes a dTP-PCR approach, which includes a combination of primers targeting a flanking region as well as from within the CGG repeat region. The primer mix was designed to amplify from the 3’ end of the *FMR1* CGG repeat region of non-modified genomic DNA. The required PCR buffer mixture and DNA polymerase were also included in the kit.

The FastFraX^TM^ SZ kit dTP-PCR assays were performed according to manufacturer’s instructions in 15-μL volumes, using 100 ng of genomic DNA per test unless otherwise stated. The assays that assessed analytic performance were conducted in duplicates or triplicates. PCR assays were carried out using the Bio-Rad C1000 (Bio-Rad). The thermal cycling conditions comprised an initial denaturation step at 95°C for 15 min, followed by 40 cycles of 99°C for 45 sec, 55°C for 45 sec, 70°C for 8 min (with an extension of 15 sec at each cycle), and then a final extension step at 72°C for 10 min.

Upon completion of the dTP-PCR, the PCR amplification products were either placed at 4°C for storage, or immediately analysed via CE. Briefly, 4 μL of each amplicon was mixed with 0.5 μL of MapMarker^®^1000-ROX (Bioventures, Inc) and 9 μL of Hi-Di™ Formamide (Applied Biosystem). The mixture was subject to denaturation at 95°C for 5 min, followed by rapid cooling to 4°C on a thermocycler. The treated mixture was then injected on an ABI 3730xL DNA Analyzer (Applied Biosystems) with a capillary length of 50 cm and loaded with POP-7 polymer. The machine settings were as follows: an injection voltage at 1200V for 18 sec, for CE at 15kV for 50 min.

### Data analysis and result interpretation

Upon completion of the CE, electropherograms were analysed with Peakscanner software (version 2.0, Applied Biosystems), following manufacturer’s instructions. First, the electropherograms were visually checked for aberrant results (e.g. contamination in the no template controls, or incorrect calling of size standard peaks). Valid sample electropherograms were then subjected to analysis of CGG repeat size. Each electropherogram was categorised into one of three possible analysis size ranges: 140 to ~300 base pair (bp), 140 to 450 bp, and 140 to >450 bp. The final peak of each sample was determined according to the assigned size range (based on signal height and/or peak morphology) and the repeat size derived by counting the first until final peak. This repeat size corresponded to the reportable size of the largest allele present in the sample. For males, only this repeat size was reported. For females, repeat size of a second, smaller allele was determined via additional analysis of the electropherogram profile pattern. Alleles determined as having greater than 200 repeats were categorically classified as having >200 repeats. Samples were then classified into the respective genotypes (NL, IM, PM, and FM) based on the reported repeat size of the largest allele, following the ACMG/EMQN guidelines.

## Results

### Genotyping and sizing accuracy

The FastFraX^TM^ SZ kit was first evaluated for its ability to provide basic size information for genotyping, using eight Coriell DNA samples representing NL, IM, PM, and FM of both male and female individuals. The typical electropherograms of each sample type were shown in S1 Fig, where one allele was reported for male samples, and two alleles reported for female samples. Based on the profiles and the peak counts, the kit provided basic information on sizes for all alleles and zygosities for the female samples, enabling the classification of these typical reference samples into the respective genotypes (i.e. NL, IM, PM, and FM) (S1 Fig.). Furthermore, the electropherograms revealed the AGG interruptions for the respective NL or PM alleles in samples (S1 Fig).

The sizing accuracy of the kit was further evaluated with an expanded panel of 29 Coriell DNA samples covering the range of NL, IM, PM and FM genotypes (Table 1). The CGG repeat size of these 29 samples had been extensively studied and reported elsewhere in at least one other study [3,13,14], in addition to data provided by Coriell Institute. For samples with a common consensus (n = 14, total 21 alleles) by the consortium study [13], the FastFraX^TM^ SZ kit reported sizing results within 3 repeats of the expected (either by Coriell or the consortium study), for all but two alleles (in NA20234 and NA20242 respectively). When the comparison was extended to those samples without a common consensus (n = 5, total 7 alleles) or those not tested (n = 10, 14 alleles) by the consortium study [13], a greater degree of variation (ranging from 1 to 43 repeats) was observed. Interestingly, when the test results were compared with the largest alleles ever reported elsewhere by any one of the studies listed in Table 1 [3,13,14], the sizes of all alleles in the 29 samples determined by the kit were within 3 repeats of the expected, save for three exceptions (Table 1). For example, NA20237 was sized by the FastFraX™ SZ kit as having 139 repeats, compared to the expected 100–104 repeats as provided by Coriell. At the same time, this sample was found by Juusola *et al*. (2012) [14] to contain an allele with 137 repeats. Similarly, NA20239 was sized by the kit as having >200 repeats. Although it differed from the expected 183–193 by at least 7 repeats, the result was consistent with the findings of Chen *et al*. (2010) [3] and Juusola *et al*. (2012) [14]. Due to the design of the FastFraX™ SZ kit, all samples with a FM allele are expected to be detected as >200 repeats. Consequently, FM alleles reported as greater than 200 repeats were considered to be in agreement with the expected data, regardless of their exact size.

Taking into consideration all the 29 samples (total 42 alleles) and all data sets presented, among the NL and IM samples, the kit reported size information within 1 repeat of the expected for all but one allele. Among the PM samples, the kit reported size information within 3 repeats of the expected for all but two alleles. When taken together with sizes reported for FM alleles, these data yielded an agreement of 92.9% (39/42 alleles) between the test and the expected result. As elaborated in the Discussion section, if the two exceptions (due to inconsistency derived from allelic instability or size mosaicism) of PM samples (NA20241, NA06896) were excluded from the tabulation, the total agreement would reach 97.5% (39/40 alleles).

### Analytic sensitivity—Limit of detection

The analytic sensitivity of the FastFraX^TM^ SZ kit was first examined with respect to its limit of detection using five Coriell DNA samples representing the male NL, IM, PM, and FM genotypes (NA06890, NA20232, CD00014, NA06892, and NA06852). The amount of genomic DNA per test (reaction) recommended by the manufacturer is 100 ng. The amount of input DNA per reaction was varied in duplicates at 25, 50, 100 and 200 ng. The limit of detection for the kit was evaluated by examining the accuracy of the sizing results of all the alleles at different levels of DNA input (S1 Table). The reduction of DNA inputs from 100 ng to 25 ng had no impact on the sizing accuracy for all alleles from the tested male samples. The results indicated that the FastFraX^TM^ SZ kit can be used with a broad range of DNA inputs (25 ng to 200 ng), without compromising performance in sizing the alleles of male samples.

Likewise, the analytic sensitivity of the kit was tested using five female Coriell DNA samples representing NL, IM, small PM, and FM genotypes (NA07538, NA20234, NA20241, NA20239 and NA07537) (S1 Table). The size of each of two alleles in the female samples was analysed individually, regardless of zygosity. The reduction of DNA inputs to 25 ng appeared to bring about considerable variation in sizing accuracy for three of the five smaller alleles of the female samples (S1 Table). Regardless, sizing accuracy for the larger alleles was not affected, and consequently, genotyping performance was not compromised. For example, at 25 ng DNA input, the smaller allele of 20 repeats in sample NA20239 was not detected. However, the larger allele of >200 repeats in the same sample remained fairly accurately sized (within 10 repeats, S1 Table). Additionally, at 50 ng DNA input, one of five smaller alleles was sized only within 6 repeats in accuracy as compared to the expected data. However, all the five larger alleles were accurately sized, again enabling correct genotyping of all the samples tested. These results indicated that the FastFraX^TM^ SZ kit can provide accurate genotyping information with DNA inputs as low as 25 ng. Still, 100 ng DNA input per test is recommended for greater sizing accuracy, particularly for the smaller alleles in female samples.

### Analytic sensitivity–detection of mosaic samples

The analytic sensitivity of the FastFraX^TM^ SZ kit was further evaluated using simulated mosaic samples. The simulated samples were created by mixing a male NL sample (NA06890, 30 CGG) with either a male PM or a male FM sample (NA20233, 120 CGG and NA04025, >200 CGG). Samples were mixed in various proportions and maintained at a total DNA input of 100 ng per reaction. The simulated samples contained 1%, 2.5%, 5%, 10%, 20%, 50% and 100% of the sample with expanded allele i.e. mosaicism level. Simulated mosaic samples containing both PM and FM alleles were also created with the amount of the FM allele varied at 1%, 2.5%, 5%, 10%, 20%, 50% and 100% of the total DNA.

With the simulated NL/PM mosaic samples, our results showed that although the less abundant PM allele could be detected as PM at as low as 2.5% of the simulated mosaicism, it was not accurately sized (S2 Table). This less abundant PM allele could be accurately sized and genotyped (within 5 repeats of the expected) only at a mosaicism level of 5% and higher. With the simulated NL/FM mosaic samples, the less abundant FM allele could be detected as large PM (>150 repeats) at a mosaicism level of 2.5%, and was detected as FM (within 2 repeats of the expected) at a mosaicism level of 5%. The less abundant FM allele could be accurately sized and genotyped only at a mosaicism level of 10% and higher. Similarly, with the simulated PM/FM mosaic samples, the less abundant FM allele could be detected as large PM (>150 repeats) at 5% and 10% mosaicism level. This less abundant FM allele could be accurately sized and genotyped at a mosaicism level of 20% and higher (S2 Table). It is worthwhile to note that even though the FM allele could not be detected at lower abundance level of 1% or 2.5% in the PM/FM mosaic, the samples were still reported to have an allelic size of at least 120 repeats due to the dominant presence of the PM allele (S2 Table).

### Analytic specificity

The primer sets of the FastFraX™ SZ kit were designed to ensure specificity to the *FMR1* CGG repeat region [10]. Analytic specificity was then assessed following an approach reported in a previous study [9], using a “non-relevant” DNA sample (NA23378) to gauge if the presence of nucleic acids distinct from CGG repeat expansions in *FMR1* would interfere with kit performance. In this study, a Coriell male DNA sample (NA23378) harboring a *DMPK* CTG repeat expansion (135–145 repeats) was added in increasing amounts (0 ng, 100 ng and 200 ng) to 100 ng of eight Coriell DNA samples representing both male and female NL, IM, PM and FM genotypes (NA20244, NA20230, NA06892 and NA06852; NA20243, NA20236, NA06894 and NA07537 respectively). The “non-relevant” DNA sample (NA23378) had been separately examined before use in the study and was verified to have a NL *FMR1* allele of 33 CGG repeats (S2 Fig).

Across all eight samples tested, the increased amounts of “non-relevant” DNA (i.e. CTG) at 100 and 200 ng did not generate any additional profile corresponding to the expected length of 135–145 CTG repeats in the electropherograms (S3 Table). All male samples under the varied condition were sized accurately well within 5 CGG repeats of the expected and, consequently, no interference with the sizing, pattern recognition and genotyping of *FMR1* CGG repeats in these samples was observed (S3 Table). Similarly, the accuracy of allele sizing and genotyping was not affected in female samples. The “interferences” (33 repeats) observed with the shorter alleles from 29 to 31 repeats in the female samples (NA20236, NA06894 and NA07537) were derived from the NL allele of 33 CGG repeats carried within NA23378 (S2 Fig). This was expected because the 33 repeats were after all relevant CGG repeats themselves (S3 Table). In all, the results demonstrated that the FastFraX^TM^ SZ kit can accurately size and genotype the *FMR1* alleles in the presence of other “non-relevant” DNA species.

### Precision–inter- and intra-assay consistency

Precision of the FastFraX^TM^ SZ kit was evaluated in two separate studies.

Intra-assay consistency was examined using 8 reference DNA samples (CD00014, NA06892, NA06852, NA07538, NA20234, NA20241, NA20239 and NA07537), covering the four *FMR1* allelic classifications and both genders. For each sample, ten PCR replicates was performed, and each of the resultant amplicon was subjected to two runs of capillary electrophoresis. Consequently, a total of 20 data points was obtained for each DNA sample tested. As shown in S4 Table, the kit produced no variation in sizing for all alleles in NL and male IM samples. Variations were observed for the larger alleles with >110 repeats, and the NL alleles in female IM, PM or FM samples, but with a coefficient of variation (CV) of no greater than 1.89%. As the kit reports all FM alleles as >200 repeats, CV parameters were not applicable for this category of the samples in the study.

Inter-assay consistency was studied using a panel of five reference DNA samples (NA06892, NA06852, NA07538, NA20241, and NA07537) representing NL, PM and FM with the PM and FM covering both male and female samples. The first part examined intra-batch repeatability by measuring consistency of kits within the same production batch. Three kits–the first, middle and final kit–from a single production assembly process were sampled. Each kit was tested with the panel of DNA samples in triplicates. The CGG repeat sizes were recorded, and variations measured in CV. The repeatability results were summarized in S4 Table. Again, no variation was observed with the NL alleles. For larger PM alleles, the maximum CV was 1.08%. For FM alleles, the CV calculation was not applicable, because the alleles were all sized consistently above the cut-off and the results were reported as >200 repeats according to the manufacturer’s instruction.

The second part of the inter-assay consistency examined inter-batch reproducibility by measuring the consistency of kits across different production batches. Data from three batches produced over a two-year period were obtained. The data set from each batch consisted of the panel of DNA samples tested in triplicate by a different operator. The reproducibility data was tabulated in S4 Table. Once more, no variation in the reported CGG repeat sizes of NL alleles was observed. For longer PM alleles, the maximum CV observed was 3.42%. Again, the CV calculation was not applicable for FM alleles as all the samples were sized consistently above the cut-off and the results reported as >200 per the manufacturer’s instruction.

### Kit performance validated with clinical samples

The performance of the FastFraX^TM^ SZ kit was ultimately evaluated using 198 archived, blinded clinical specimens that were previously characterized for their *FMR1* CGG repeat size using a combination of flanking PCR [16], TP-PCR and/or Southern blot analysis [17]. These clinical specimens were genomic DNA derived from patient whole blood samples from a cohort with intellectual disabilities. The kit sized 90.40% of the clinical samples well within 1 repeat of the reference results, and 98.48% well within 5 repeats of the reference results (Table 2). Based on the size reported by the kit, the samples were then classified according to their respective genotype of NL (n = 155), IM (n = 4), PM (n = 21) and FM (n = 18). This was 100% concordant with the classification determined by the reference methods (Table 3). Thus, the overall performance of the kit was demonstrated with 100% clinical specificity and sensitivity (95% CI: 97.64% to 100% for specificity; 91.03% to 100% for sensitivity, respectively). The findings also indicated a kit performance of 100% positive predictive value (PPV) and negative predictive value (NPV).

**Table 2:** Repeat size concordance of clinical samples using the FastFraX^TM^ SZ kit in comparison with previously characterised methods from Mundhofir *et al*.^†^ [17].

Genotype from Mundhofir *et al*. [17]	Repeat Size Concordance	
	Within 5 repeats (±5)	Within 1 repeat (±1)
*Males*		
NL	79 / 79	77 / 79
IM	4 / 4	3 / 4
PM (<110 repeats)	4 / 5	0 / 5
PM (≥110 repeats)	2 / 2	2 / 2
FM	8/8*	8/8*
*Females*		
NL	76 / 76	75 / 76
IM	-	-
PM (<110 repeats)	10 / 10	3 / 10
PM (≥110 repeats)	2 / 4	1 / 4
FM	10/10*	10/10*
**Total Agreement**	**195/198 (98.48%)**	**179/198 (90.40%)**

† Taking into consideration alleles not detected by the original reference approach of Mundhofir *et al*.[17], but identified by supplemental verification for allelic variation using the method of Rajan-Babu *et al*. (2015) [19] (see also Table 4).

* Full mutation samples are considered concordant as long as they were reported as having >200 repeats.

**Table 3:** Classification of clinical samples using the FastFraX^TM^ SZ kit in comparison with previously characterised methods from Mundhofir *et al*. [17]

			Classification from Mundhofir *et al*. [17]				
			Non-Expanded		Expanded		
			NL	IM	PM	FM	
**Classification using FastFraX**^**TM**^ **SZ kit**	**Non-Expanded**	**NL**	155^TN^	0^TN^	0^TN^	0^FN^	**Negative Predictive Value = TN/(TN+FN)** 100.00%
		**IM**	0^TN^	4^TN^	0^TN^	0^FN^	
	**Expanded**	**PM**	0^TN^	0^TN^	21^TN^	0^FN^	**Positive Predictive Value = TP/(TP+FP)** 100.00%
		**FM**	0^FP^	0^FP^	0^FP^	18^TP^	
			**Clinical Specificity = TN/(TN+FP)** 100.00% (95% CI: 97.64–100.00%)		**Clinical Sensitivity = TP/(TP+FN)** 100.00% (95% CI: 91.03–100.00%)		

TN: True Negative FN: False Negative TP: True Positive FP: False Positive

### Detection of clinical samples with mosaicism or size instability

Among the 198 clinical specimens, 14 samples had been determined by a combination of other methods [16,17,19] to be harboring PM and FM *FMR1* alleles of various sizes (i.e. size mosaicism and size instability). These samples and their test results were summarized and shown in Table 4.

**Table 4:** Repeat size of selected mosaic clinical samples using the FastFraX^TM^ SZ kit in comparison with previously characterised methods from Mundhofir *et al*. [17].

Assigned ID	Category	No. of CGG Repeats	
		Expected from Mundhofir *et al*.[17]	FastFraX^TM^ SZ kit
*Males*			
14	PM / SI	86	-
		103	108
		(113)	-
20	PM / SI	86	-
		166	-
		(178)	182
12	FM / SM	(130)	-
		>200	>200
15	FM / SM	80	-
		103	-
		(179)	-
		>200	>200
52	FM / SM	(127)	-
		>200	>200
54	FM / SM	(123)	-
		>200	>200
58	FM / SM	71	-
		>200	>200
199	FM / SM	(132)	-
		>200	>200
*Females*			
70	PM / SI	30	30
		152	-
		(192)	192
200	PM / SI	30	30
		150	-
		(190)	199
59	FM / SM	44	33
		(106)	-
		>200	>200
135	FM / SM	29	29
		139	-
		>200	>200
138	FM / SM	29	29
		223	>200
		446	-
		769	-
139	FM / SM	29	29
		110	-
		222	>200
		441	-

SM: Size mosaic SI: Indication of size instability within the sample

(): Alleles not detected by the original reference approach of Mundhofir *et al*.[17] but identified by supplemental verification for allelic variation using the method of Rajan-Babu *et al*. (2015) [19]_._

Although the FastFraX^TM^ SZ kit was able to provide size information for both alleles in typical female heterozygotes (S1 Fig), the electropherograms generated by the kit were inadequate for reporting multiple alleles present in the clinical samples with size mosaicism and size instability. Instead, the kit detected and sized only the allele with the largest CGG repeat expansion, particularly with male samples (Table 4). For example, a male PM sample with size instability (PM/SI No. 14) that was determined by the combination of methods to contain two alleles of 86 and 103 repeats, was sized by the FastFraX^TM^ SZ kit as 108 repeats, which was well within 5 repeats of the larger allele (103 repeats). Further, a male FM size mosaic sample (FM/SM No. 15) that was reported by the combination of methods to contain alleles of 80, 103, and >200 repeats, was sized by the SZ kit as >200 repeats. For female samples, the presence of the smaller NL allele could still be identified, and thus resolution of zygosity was not affected. However, a consistent pattern of sizing only the largest allele among the expanded alleles was observed, in both FM size mosaic samples as well as in PM samples with size instability (Table 4).

In summary, the FastFraX^TM^ SZ kit detects the mosaic samples and samples with size instability by detecting the largest allele, which facilitates the accurate genotyping of the tested samples. However, it will not indicate mosaicism because the kiy does not provide information for all the alleles present in the samples.

## Discussion

The present study was carried out to validate the use of a newly available kit in sizing CGG repeats in the *FMR1* gene. The design principle of this assay has been reported elsewhere [10] and briefly, it is a TP-PCR based test directly targeting the CGG triplet repeats. The new assay differs from the existing approach of Chen *et al*. [3] in that it provides information on size and AGG interruptions without the need for a set of gene-specific primers.

This kit performed well in both accurately classifying representative samples according to the four allelic genotypes (based on samples from Coriell Institute), and in indicating the AGG interruption pattern (S1 Fig). The kit’s excellent genotyping performance is due to its ability in generating accurate sizing data. This was established by testing the kit with 29 samples from Coriell Institute. The FastFraX^TM^ SZ kit sized 39/42 alleles well within 3 repeats of the reference data; producing an agreement of 92.9% with the Coriell reference and/or data published by other studies (Table 1). It must be noted here that the evaluation included not just samples found to have consensus by a consortium study [13] (n = 14), but also those without consensus by the consortium study [13] (n = 5), and those reported elsewhere with different sizes by other studies [3,14] (n = 10). Consequently, greater variation in sizing data was obtained for the latter two groups of samples. The variation in repeat sizes observed included not just those derived from testing methods (test kit), but also those caused by mosaicism or allele instability inherent in the samples. In particular, NA06896 was found by the FastFraX^TM^ SZ kit to contain a larger allele (183 repeats) than the size range (95–140 repeats) provided by Coriell Institute, and had been determined by Chen *et al*. [3] to contain a cluster of alleles of varying sizes, including those beyond 200 repeats. In contrast, in the case of NA20241, the kit detected an allele far larger than that detected by Juusola *et al*. [14] which uses a method identical to Chen *et al*. [3]. Accordingly, we are of the opinion that such variations may not be solely due to differences in test methods. Sample allelic instability or somatic variation could have contributed to the non-consensus results reported in the consortium study [13] and the disagreement in results between the three approaches compared here. When sample allelic instability and somatic variation were taken into consideration and the two contradictory findings excluded from the tabulation, the agreement in sizes of all alleles that was within 3 repeats between the test and reference data increased to 97.5% (39/40 alleles, Table 1). The single remaining discordant allele (female NL sample NA20234) deviated just slightly more than 3 repeats from the reference. This sample would have tested as “non-expanded” by the screening assay [9], and would thus have been excluded from testing with the FastFraX^TM^ SZ kit. Nevertheless, such variation in sizing did not affect the performance of the kit in classifying the clinical samples tested (N = 198) accurately into the respective allelic classes, as compared to the reference approach (Table 3). Essentially, the results of the present study showed that the kit detected all clinical cases of PM and FM accurately, yielding a 100% in both clinical sensitivity and specificity (95% CI: 91.03% to 100.00% and 95% CI, 97.64% to 100.00% respectively) (Table 3).

The utility of TP-PCR in overcoming the key limitations of conventional flanking PCR strategies has been well established. Conventional flanking PCR is unable to detect large alleles or larger alleles in specimens with multiple alleles, such as females or mosaics [1]. Recently, the advantage of TP-PCR was further demonstrated through the discovery that TP-PCR could detect mosaic samples effectively, by simply coupling with melt curve analysis [9,10]. In this regard, the present study added further supporting data to the existing literature on the advantages of using TP-PCR without an opposing pair of gene-specific primers. Specifically, the present study with the simulated mosaic samples indicated that the FastFraX^TM^ SZ kit readily detected the larger alleles even at low abundance levels of 2.5–5.0% in all the tested samples (S2 Table). Furthermore, the kit demonstrated capability in detecting PM/NL and FM/NL simulated mosaics at 5% mosaicism level, and FM/PM mosaics at 20% mosaicism level, to the accuracy of within 5 repeats of the expected CGG repeat size (S2 Table). Separately, the present study with the clinical mosaic samples (including samples with allelic instability) also indicated that the kit performance in mosaic detection is readily achievable at clinical settings. The larger/largest alleles in the clinical mosaic samples or samples with allele instability could be detected with an accuracy of within 5 repeats when compared with those generated by the reference methods, with the exception of three samples (Nos. 20, 70 and 200). These three samples demonstrated larger disagreements in repeat size (approaching 50 repeats). When results for these three samples were further compared with those additional alleles detected by a third test using a methylation-specific method [19], the differences were significantly narrowed down (Table 4). Consequently, it was justifiably concluded that the deviation in size observed for the three samples was due to the increased sensitivity of the SZ kit in detecting the largest allele species in mosaic samples, as compared to the reference approach using gene-specific flanking PCR.

It is necessary to highlight that although the FastFraX^TM^ SZ kit resolved zygosity in female samples, detected all clinical mosaics (or size instabilities) in both males and females, and enabled correct categorizing of the respective genotypes as per either ACMG or EMQN guidelines; it did not provide specific information on CGG repeat size when multiple alleles were present in the sample. This trade-off was due to the exclusion of the gene-specific primers in the kit; consequently, only the electropherograms of triplet repeat-primed amplicons were available for analyses. While detection of the additional allele(s) would be helpful in indicating mosaicism, its clinical utility is limited if there is no accompanying information on methylation status. Ultimately, the clinical implications of size somatic mosaicism in *FMR1* PM or FM alleles are linked to *FMR1* mRNA and FMRP expression, which are both correlated with the percent of methylation [20]. Thus, to clarify severity of the clinical phenotype, it is critical to verify the methylation status of all samples found to contain large PM or FM alleles. Nevertheless, the FastFraX^TM^ SZ kit is more suitable for accurately categorizing samples into the respective allelic forms. Where methylation status is of concern (particularly for mosaic samples), the kit can be complemented or replaced by methylation-specific testing. In other words, it is important to use the appropriate kit to generate information that is relevant to the intended testing purpose.

Clinical performance aside, the FastFraX^TM^ SZ kit was found easy to use, with fair consistency and robustness. Although the recommended DNA input is 100 ng, the kit performed reasonably well at as low as 25 ng in its limit of detection tests. All in all, the CVs for intra, inter assay variations were well <2%, with a CV for reproducibility at <0.39% with only one sample being the exception of 3.4%. These parameters assured that the kit performances were easily obtainable at the user’s end. Regardless, and as emphasized above, the kit addressed the specific testing need for accurately categorizing samples into the respective genotypes, and is a key addition to a workflow to precisely determine the CGG repeat size and *FMR1* methylation status of the samples being interrogated.

## Supporting information

S1 Fig CE profiles of well-characterised male and female samples from Coriell Cell Repositories, representing the NL, IM, PM, and FM genotypes. Coriell Cell Repositories catalogue IDs, and the repeat size reported by the FastFraX^TM^ SZ kit are indicated. CGG repeat structures are indicated in brackets, where ‘+’ represents an AGG interruption.
(TIF)

S2 Fig CE profile of NA23378 sample with a “non-relevant expansion”, but carries a normal 33 *FMR1* CGG repeats. (TIF)

S1 Table Limit of detection of the FastFraXTM SZ kit, using genomic DNA samples from Coriell Cell Repositories. (DOCX)

S2 Table Mosaicism detection of the FastFraXTM SZ kit, using genomic DNA samples from Coriell Cell Repositories. (DOCX)

S3 Table Analytical specificity of the FastFraXTM SZ kit, using genomic DNA samples from Coriell Cell Repositories. A “non-relevant” DNA sample (NA23378) is added in increasing amounts of 100 ng and 200 ng.
(DOCX)

S4 Table Precision of the FastFraXTM SZ kit, using genomic DNA samples from Coriell Cell Repositories. (DOCX)
