# Supplementary figures and images for: Validation of a commercially available test that enables the quantification of the numbers of CGG trinucleotide repeat expansion in FMR1 gene
Source: PLoS One. 2017 Mar 9;12(3):e0173279. doi: 10.1371/journal.pone.0173279 (PMC5344422; doi:10.1371/journal.pone.0173279)

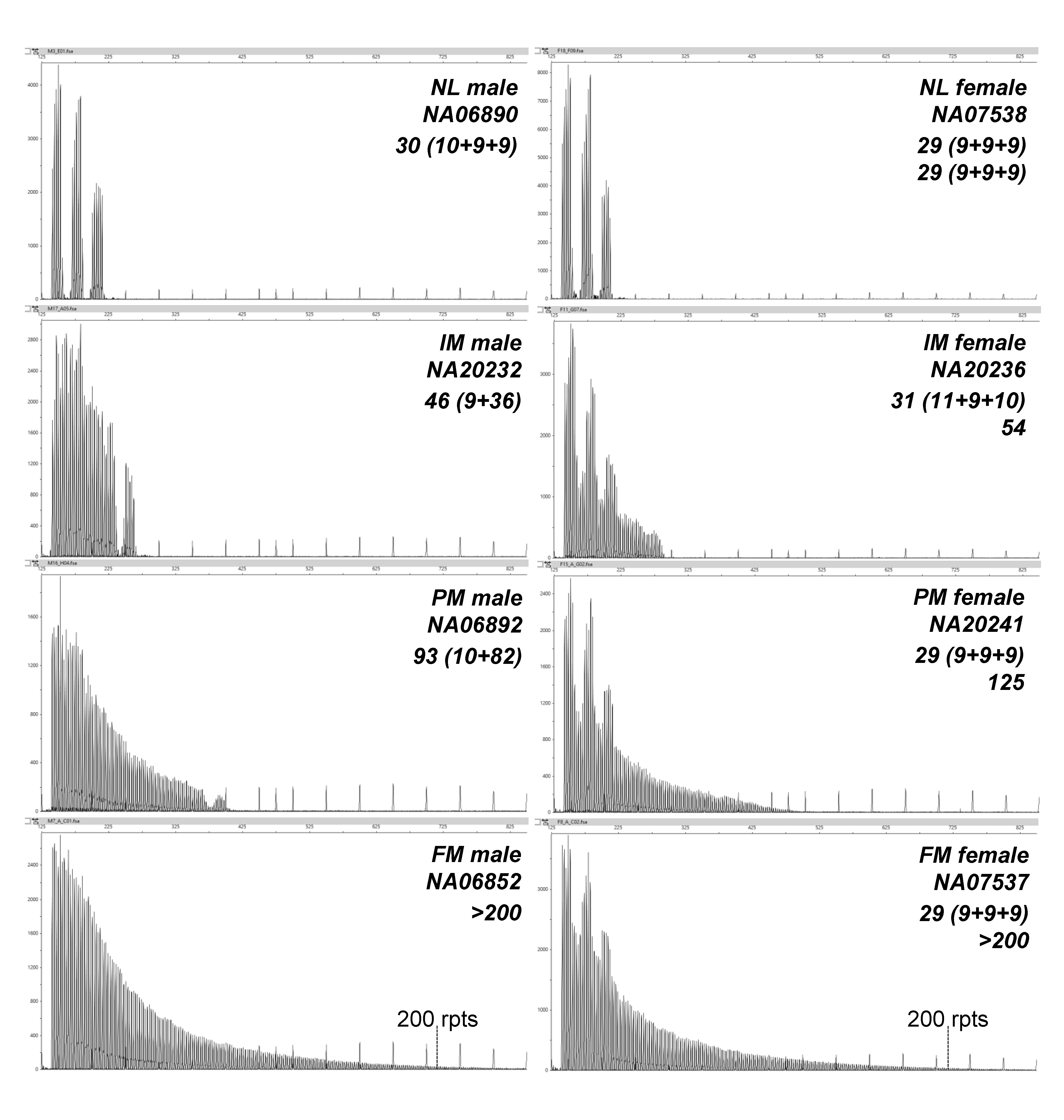

Supplement: S1 Fig — Coriell Cell Repositories catalogue IDs, and the repeat size reported by the FastFraXTM SZ kit are indicated. CGG repeat structures are indicated in brackets, where ‘+’ represents an AGG interruption. (TIF) [file pone.0173279.s001.tif]

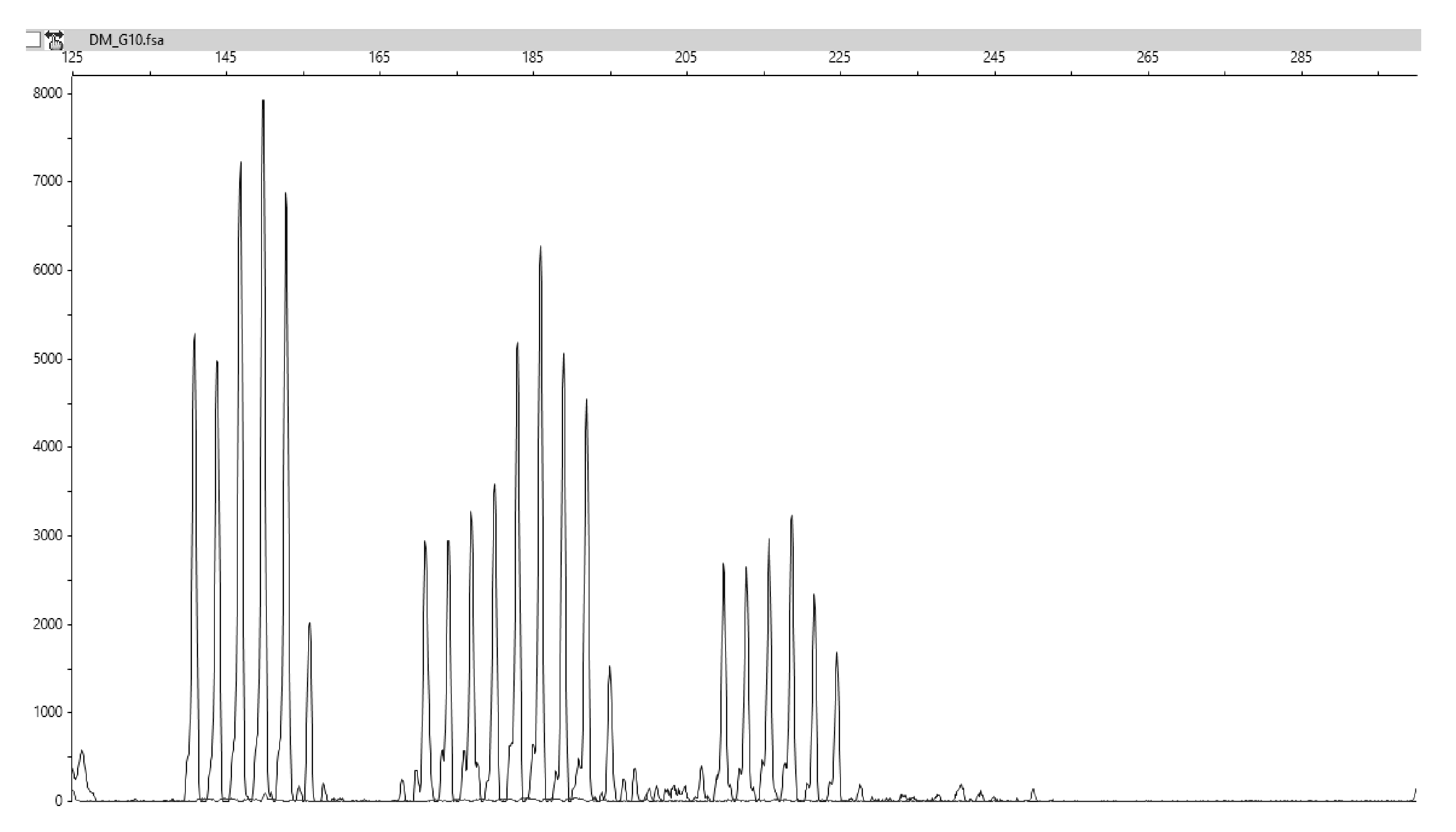

Supplement: S2 Fig — (TIF) [file pone.0173279.s002.tif]
