# Supplementary material for: Validation of a commercially available test that enables the quantification of the numbers of CGG trinucleotide repeat expansion in FMR1 gene
Source: PLoS One. 2017 Mar 9;12(3):e0173279. doi: 10.1371/journal.pone.0173279 (PMC5344422; doi:10.1371/journal.pone.0173279)
Supplement: S3 Table — A “non-relevant” DNA sample (NA23378) is added in increasing amounts of 100 ng and 200 ng. (DOCX) [file pone.0173279.s005.docx]

**S3 Table. Analytical specificity of the FastFraX^TM^ SZ kit, using genomic DNA samples from Coriell Cell Repositories. A “non-relevant” DNA sample (NA23378) is added in increasing amounts of 100 ng and 200 ng.**

| **Coriell Sample ID** | **Genotype** | **No. of CGG Repeats** | | | | | | | | |
| --- | --- | --- | --- | --- | --- | --- | --- | --- | --- | --- |
|  |  | **Expected* (X)** | | **FastFraX^TM^ SZ kit (Y)** | | |  | **Difference (Y-X)** | | |
|  |  |  |  | **0ng** | **100ng** | **200ng** |  | **0ng** | **100ng** | **200ng** |
| *Males* | | | | | | | | | | |
| NA20244 | NL |  | 41 | 41 | 41 | 41 |  | 0 | 0 | 0 |
| NA20230 | IM |  | 54 | 54 | 54 | 54 |  | 0 | 0 | 0 |
| NA06892 | PM |  | 93 | 93 | 93 | 93 |  | 0 | 0 | 0 |
| NA06852 | FM |  | >200 | >200 (215) | 196 | 199 |  | N/A | -4 | -1 |
| *Females* | | | | | | | | | | |
| NA20243 | NL | Allele 1 | 29 | 29 | 29 | 29 |  | 0 | 0 | 0 |
|  |  | Allele 2 | 41 | 41 | 41 | 41 |  | 0 | 0 | 0 |
| NA20236 | IM | Allele 1 | 31 | 31 | 33 | 33 |  | 0 | +2 | +2 |
|  |  | Allele 2 | 54 | 54 | 54 | 54 |  | 0 | 0 | 0 |
| NA06894 | PM | Allele 1 | 30 | 30 | 30 | 33 |  | 0 | 0 | +3 |
|  |  | Allele 2 | 82 | 82 | 82 | 82 |  | 0 | 0 | 0 |
| NA07537 | FM | Allele 1 | 29 | 29 | 29 | 33 |  | 0 | 0 | +4 |
|  |  | Allele 2 | >200 | >200 (215) | >200 (204) | >200 (202) |  | N/A | N/A | N/A |

* Expected based on result obtained using optimal assay conditions, following manufacturer’s instructions.

N/A: Not applicable, as the FastFraX^TM^ SZ kit reports all FM as >200 repeats. Hence, difference in repeat size is not calculated.
