# Supplementary material for: Validation of a commercially available test that enables the quantification of the numbers of CGG trinucleotide repeat expansion in FMR1 gene
Source: PLoS One. 2017 Mar 9;12(3):e0173279. doi: 10.1371/journal.pone.0173279 (PMC5344422; doi:10.1371/journal.pone.0173279)
Supplement: S4 Table — (DOCX) [file pone.0173279.s006.docx]

**S4 Table. Precision of the FastFraX^TM^ SZ kit, using genomic DNA samples from Coriell Cell Repositories.**

| **Coriell Sample ID** | **Genotype** | **No. of CGG Repeats** | | | | | | | | | | | | |
| --- | --- | --- | --- | --- | --- | --- | --- | --- | --- | --- | --- | --- | --- | --- |
|  |  | **Expected* (X)** | | **FastFraX^TM^ SZ kit** | | | | | | | | | | |
|  |  |  |  | **Intra-assay variation (n=20)** | | |  | **Inter-Assay Variation** | | | | | | |
|  |  |  |  |  |  |  |  | **Intra-batch Repeatability (n=9)** | | |  | **Inter-batch Reproducibility (n=26)** | | |
|  |  |  |  | **Avg.** | **SD** | **CV** |  | **Avg.** | **SD** | **CV** |  | **Avg.** | **SD** | **CV** |
| *Males* | | | | | | | | | | | | | | |
| CD00014 | IM |  | 56 | 56 | 0 | 0 |  | *Not tested* | | |  | *Not tested* | | |
| NA06892 | PM |  | 93 | 93 | 0 | 0 |  | 93.44 | 0.53 | 0.56 |  | 93.15 | 0.37 | 0.39 |
| NA06852 | FM |  | >200 | >200 (243) | N/A | N/A |  | >200 (233.89) | N/A | N/A |  | >200 (236.42) | N/A | N/A |
| *Females* | | | | | | | | | | | | | | |
| NA07538 | NL | Allele 1 | 29 | 29 | 0 | 0 |  | 29 | 0 | 0 |  | 29 | 0 | 0 |
|  |  | Allele 2 | 29 | 29 | 0 | 0 |  | 29 | 0 | 0 |  | 29 | 0 | 0 |
| NA20234 | IM | Allele 1 | 26 | 26.2 | 0.41 | 1.57 |  | *Not tested* | | |  | *Not tested* | | |
|  |  | Allele 2 | 46 | 46 | 0 | 0 |  |  |  |  |  |  |  |  |
| NA20241 | PM | Allele 1 | 29 | 29 | 0 | 0 |  | 29 | 0 | 0 |  | 29 | 0 | 0 |
|  |  | Allele 2 | 125 | 124.15 | 2.35 | 1.89 |  | 122 | 1.32 | 1.08 |  | 124.69 | 4.26 | 3.42 |
| NA20239 | PM | Allele 1 | 20 | 20.1 | 0.31 | 1.53 |  | *Not tested* | | |  | *Not tested* | | |
|  |  | Allele 2 | >200 | 202.7 | N/A | N/A |  |  |  |  |  |  |  |  |
| NA07537 | FM | Allele 1 | 29 | 29 | 0.45 | 1.58 |  | 29 | 0 | 0 |  | 29 | 0 | 0 |
|  |  | Allele 2 | >200 | >200 (238.8) | N/A | N/A |  | >200 (219.56) | N/A | N/A |  | >200 (226.23) | N/A | N/A |

* Expected based on result obtained using optimal assay conditions, following manufacturer’s instructions. Individual allele information may differ from that provided by Coriell Institute, but are supported by data from other studies [3,14].

N/A: Not applicable, as the FastFraX^TM^ SZ kit reports all FM as >200 repeats. Hence, standard deviation and coefficient of variation are not calculated.
